# Predicting rehospitalization within 2 years of initial patient admission for a major depressive episode: a multimodal machine learning approach

**DOI:** 10.1038/s41398-019-0615-2

**Published:** 2019-11-11

**Authors:** Micah Cearns, Nils Opel, Scott Clark, Claas Kaehler, Anbupalam Thalamuthu, Walter Heindel, Theresa Winter, Henning Teismann, Heike Minnerup, Udo Dannlowski, Klaus Berger, Bernhard T. Baune

**Affiliations:** 1 Discipline of Psychiatry, School of Medicine, University of Adelaide, Adelaide, Australia; 2 Department of Psychiatry, University of Münster, Münster, Germany; 3 Interdisciplinary Centre for Clinical Research (IZKF), University of Münster, Münster, Germany; 4 Centre for Healthy Brain Ageing, UNSW, Sydney, Australia; 5 Institute of Clinical Radiology, University of Münster, Münster, Germany; 6 Institute of Clinical Chemistry and Laboratory Medicine, University Medicine Greifswald, Greifswald, Germany; 7 Integrated Research Biobank, University Medicine Greifswald, Greifswald, Germany; 8 Institute of Epidemiology and Social Medicine, University of Münster, Münster, Germany; 9 Department of Psychiatry, Melbourne Medical School, The University of Melbourne, Melbourne, Australia; 10 The Florey Institute of Neuroscience and Mental Health, The University of Melbourne, Melbourne, Australia

**Keywords:** Human behaviour, Prognostic markers

## Abstract

Machine learning methods show promise to translate univariate biomarker findings into clinically useful multivariate decision support systems. At current, works in major depressive disorder have predominantly focused on neuroimaging and clinical predictor modalities, with genetic, blood-biomarker, and cardiovascular modalities lacking. In addition, the prediction of rehospitalization after an initial inpatient major depressive episode is yet to be explored, despite its clinical importance. To address this gap in the literature, we have used baseline clinical, structural imaging, blood-biomarker, genetic (polygenic risk scores), bioelectrical impedance and electrocardiography predictors to predict rehospitalization within 2 years of an initial inpatient episode of major depression. Three hundred and eighty patients from the ongoing 12-year Bidirect study were included in the analysis (rehospitalized: yes = 102, no = 278). Inclusion criteria was age ≥35 and <66 years, a current or recent hospitalisation for a major depressive episode and complete structural imaging and genetic data. Optimal performance was achieved with a multimodal panel containing structural imaging, blood-biomarker, clinical, medication type, and sleep quality predictors, attaining a test AUC of 67.74 (*p* = 9.99^−05^). This multimodal solution outperformed models based on clinical variables alone, combined biomarkers, and individual data modality prognostication for rehospitalization prediction. This finding points to the potential of predictive models that combine multimodal clinical and biomarker data in the development of clinical decision support systems.

## Introduction

Relapse rates in specialized mental healthcare settings are high in patients with major depressive disorder (MDD) (60% after 5 years, 67% after 10, and 85% after 15)^1^. To better predict relapse, previous research has predominantly focused on the presence and magnitude of clinical symptoms, including residual depressive symptoms^2^, illness severity^3^, number of prior episodes^4^, age of onset^4^, and comorbid personality disorders^5^. Other studies have explored group level associations between biomarkers and relapse, observing smaller hippocampal volumes^6^, higher levels of post-treatment glucocorticoids^7^, high cortisol response on the combined dexamethasone-CRH test^8^, as well as catecholamine and tryptophan depletion^9^. Such findings have been beneficial in constructing modality specific aetiological hypotheses as well as sign posts for relapse in clinical practice. However, the elucidation of clinically meaningful predictors of relapse is contingent on the construct validity of the prediction outcome and the size of the analysed sample. Some of these studies identifying biomarkers as predictors of relapse have used self-reported relapse into a new depressive episode and samples of less than 50 patients^7,10^. Whether patients have truly relapsed into a new depressive episode or merely have a treatment refractory illness is unknown. These observations may help to explain the lack of empirically validated relapse prediction tools and subsequent intervention strategies for relapse prevention^10^.

To overcome this blind spot in patient care, studies of increased size that focus on a well-defined outcome such as rehospitalization are needed to identify clinically robust predictors of this illness trajectory. To facilitate this, a longitudinal, multimodal sample of clinically diagnosed inpatients is required. The BiDirect depression cohort provides such a sample^11^. In addition, multimodal, multivariate modelling techniques that prognosticate individually, rather than at the group level are needed given the heterogeneous nature of MDD and its illness trajectories^12,13^.

Recent work has shown that machine learning (ML) models are well suited to problems of this nature, demonstrating their efficacy in drug response and functional outcome prediction in MDD^14,15^, while other studies using neuroimaging modalities have shown similar success for ML-based MDD diagnostics^16^. Interestingly, no studies have applied ML models to relapse prediction; thus, the utility of differing data modalities for relapse prediction remains poorly understood. Regarding illness trajectory modelling, one previous work from Schmaal et al.^17^ combined neuroimaging and clinical data to predict MDD remission trajectories with moderate success (accuracy = 69–73%). Further, Koutsouleris et al.^15^ used both neuroimaging and clinical data to predict functional outcomes in recent onset MDD (balanced accuracy = 70.3%). However, studies employing a combination of neuroimaging markers with biomarkers from different modalities as well as clinical data are lacking up until now.

Given the apparent gap in multimodal studies of outcome prediction in MDD, we have combined a range of clinical and biomarker predictors that have shown significant associations to MDD in previous works but are yet to be used for ML-based rehospitalization trajectory modelling. Modalities used included clinical^18^, blood biomarker^19–21^, structural imaging^16^, electrocardiography^22^, genetic^23^, cognitive^24^, nutritional^25^, sleep^26^, and exercise^27^. Using these modalities, we predicted rehospitalization in a cohort of patients within 2 years of initial hospitalization for an acute episode of MDD.

## Materials and methods

### Dataset description

The BiDirect study is an ongoing study of (a) patients, hospitalized for an acute episode of major depression at time of recruitment, (b) population controls randomly drawn from the register of the city of Münster^11^, and (c) patients 3 months after an acute coronary event or myocardial infarction. Examination of all participants included a computer-assisted face-to-face interview of socio-demographic characteristics and medical history as well as an extensive psychiatric assessment (Supplementary Information 1.1). Only patients in the depression cohort were used in this analysis.

At baseline, 999 MDD patients were recruited and 684 completed their 2-year follow-up and provided their rehospitalization status. Specifically, patients were asked, have you had at least one or more re-admissions to hospital for an acute depressive episode since the initial examination? Patient response was recorded by study assistants. As our aim was to assess both multi and unimodal prediction models of reported rehospitalization, a requirement for inclusion was complete imaging and genetic data. Twenty-nine patients had incomplete genetic data while 294 had incomplete imaging data. In addition, 14 participants were excluded due to poor MRI quality, leaving a final sample of 380 participants (rehospitalized: yes = 102, no = 278). See Table 1 for sociodemographic characteristics. In this final sample, 87.9% (334/380) of patients were taking some form of antidepressant medication at their baseline assessment, 40.3% (153/380) were taking an antipsychotic, while 92.6% (352/380) were taking some form of psychotropic medication (see Table 2).

**Table 1:** Summary statistics for the final study sample

	Mean	SD	Min	Max		Mean	SD	Min	Max	*P*
Rehospitalized? Yes (*n* = 102)					Rehospitalized? No (*n* = 278)					
Sex (m/f)					Sex (m/f)					
(*n* = 43/59)					(*n* = 108/170)					
Age					Age					
(*n* = 102)	49.03	7.32	34.96	63.96	(*n* = 278)	49.91	7.38	35.15	65.37	0.3
HAM-D total					HAM-D total					
(*n* = 101)	15.33	6.59	0.00	27.00	(*n* = 278)	12.71	6.33	0.00	33.00	<0.01
CES-D total					CES-D total					
(*n* = 102)	31.30	12.97	1.00	56.00	(*n* = 276)	25.40	11.50	0.00	48.00	<0.01
Total inpatient episodes					Total inpatient episodes					
(*n* = 101)	2.06	2.00	0.00	10.00	(*n* = 274)	1.42	0.90	0.00	6.00	<0.01

Means, standard deviations (SD), minimal (Min), and maximal (Max) values are presented. Significance testing between groups was conducted with independent samples *t*-tests. “Total inpatient episodes” includes the baseline assessment inpatient episode as well as all previous inpatient episodes. *HAM-D total* total score for the first 17 items of the HAM scale; *CES-D total* total score with the inversion of positive items 4, 8, 12, and 16 taken into account

**Table 2:** Percentage proportions and total counts for psychotropic medication use in each rehospitalization outcome group

Medication	Rehospitalized? Yes (*N* = 278)	Rehospitalized? No (*N* = 102)	*P*
Selective serotonin reuptake inhibitors	29.50% (*N* = 82)	22.55% (*N* = 23)	0.18
Beta blocking agents	18.71% (*N* = 52)	10.78% (*N* = 11)	0.07
Non-selective monoamine reuptake inhibitors	10.43% (*N* = 29)	13.73% (*N* = 14)	0.37
Other antidepressants	57.91% (*N* = 161)	71.57% (*N* = 73)	0.06
Benzodiazepines	30.58% (*N* = 85)	53.92% (*N* = 55)	<0.01
Butyrophenone derivates	3.96% (*N* = 11)	8.82% (*N* = 9)	0.06
Diazepines, oxazepines, thiazepines, oxepines	24.10% (*N* = 67)	41.18% (*N* = 42)	<0.01
Lithium	2.52% (*N* = 7)	3.92% (*N* = 4)	0.47
Other antipsychotics	6.47% (*N* = 18)	17.65% (*N* = 18)	0.01

Significant differences between groups were assessed using chi-square tests

### Predictor modalities

#### Clinical

Detailed information regarding socio-demographic and socio-economic status, lifetime medical diagnoses, current medication use, healthcare utilization, insurance status, lifestyle and risk behaviour (e.g., diet, physical activity, alcohol consumption, smoking status), and perceived health state was collected via a computer-assisted interview. A combination of individual items as well as total scores were included from the Hamilton Depression Rating Scale (HAM-D), the Hamilton Anxiety Rating Scale (HAM-A), the Center for Epidemiologic Studies Depression Scale (CES-D), the Inventory of Depressive Symptomatology (IDS), the International Physical Activity questionnaire (IPAQ), and the Food Frequency Questionnaire (FFQ) (Supplementary Information 1.1 and 1.2). In addition, we included measures from a cognitive functioning module and several self-report measures (Supplementary Information 1.3). In total, 208 clinical and demographic predictors were included.

#### Structural imaging

We included imaging data derived from structural magnetic imaging sequences (3D-T1). To reduce the size of the predictor space, we a priori selected 15 regions that have been shown to be significantly associated with MDD in previous ENIGMA meta-analyses^6,28^. Selected regions included right and left mean hippocampal volume, cortical thickness of the bilateral medial orbitofrontal cortex (OFC), fusiform gyrus, insula, rostral anterior and posterior cingulate cortex and unilaterally in the left middle temporal gyrus, right inferior temporal gyrus and the right caudal anterior cingulate cortex (Supplementary Information 1.4).

#### Serum and genetic markers

Our serum biomarker panel consisted of 10 measures of high sensitive C-reactive protein^19^, free triiodothyronine, thyroxine, thyroid-stimulating hormone^29–31^, 17 beta-estradiol^32,33^, sex hormone-binding globulin, testosterone, the free androgen index^34–36^, total cholesterol^37^, and high-density lipoprotein cholesterol^38^. Due to the correlations between genetic variants shared across psychiatric traits and common comorbidities between psychiatric disorders^39,40^, we also included seven polygenic risk scores (PGRS) with a *p* value threshold of 0.5, for MDD, anxiety, Alzheimer’s, anorexia, autism spectrum disorder, bipolar, and schizophrenia (Supplementary Information 1.5–1.6 and Supplementary Information 2.1–2.6). In addition, we also assessed PGRS with *p* value thresholds of 0.05 and 0.01 (Supplementary Information 3.2).

#### Cardiovascular

We determined cardiovascular and general health status through the assessment of different cardiovascular markers. First, we measured weight (without shoes and heavier clothes), height, and waist circumference. Following, we used bioelectrical impedance measurements (Body Impedance Analyzer BIA 2000-S, Data Input GmbH) including the determination of body fat and water, extracellular mass, body cell mass, and basic metabolic rate to assess general markers of body composition. In addition, a measurement of a standard three-channel electrocardiogram (ECG) was performed (Supplementary Information 1.7). From these assessments we included the following seven predictors: heart rate (beats per minute), body mass index (BMI), extracellular mass to body cell mass (ECM/BCM ratio), basic metabolic rate, corrected body fat (kg), total body water (kg), and lean body mass (kg). In total, 247 predictors were included in the analysis (see Supplementary Table 1 for all predictors).

### ML pipeline

For our first set of models we entered all predictor modalities into our pipeline for consideration. To ensure the unbiased approximation of the model’s generalizability to new patients, we trained and tested all models using repeated nested cross-validation in a pipeline to prevent information leaking between patients used for training and validation. In the inner cross validation loop, we conducted imputation, standardization, feature selection, hyperparameter optimization, and the fitting of a linear support vector machine (SVM).

To begin, we imputed predictors using multivariate imputation of chained equations with the 10 nearest predictors used in the imputation process. No predictors entered into the pipeline had more than 20% missing data. Following, all variables were scaled to have a mean of zero and a standard deviation of one. Next, we used the elastic net, a form of penalized logistic regression to select a final subset of variables for prediction. This approach shrinks the coefficients of highly correlated predictors towards each other while removing irrelevant predictors from the model^41,42^. This process was completed simultaneously with an exhaustive grid search to tune model hyperparameters. For the elastic net, the parameters alpha (the amount of penalization) and the l1 ratio (mixing parameter between the l1 and l2 norms) were tuned for predictor selection. For the l1 ratio parameter we searched the values *λ*∈{0.1, 0.5, 0.7, 0.9, 0.95, 0.99}, with values closer to 1 representing the l1 norm. For alpha, we searched *a*∈{0.1, 0.2…,1.0}. For the SVM, we tuned four values of the regularization parameter *C*∈{0.001, 0.01, 0.1, 1.0}. To accommodate for the class imbalance in the outcome, each *C* value was weighted by the inverse percentage proportion of each class label (rehospitalized: yes = 102, no = 278). This approach increases the penalty for misclassifying the minority class (rehospitalized) given its relative scarcity. Finally, we used Platt scaling to calibrate the probability estimates for the SVMs binary predictions^43,44^. The set of predictors and hyperparameters that maximized area under the curve on the receiver operator characteristic (AUC) were selected in the pipeline. See Supplementary Information 1.8–1.8.7 for further details on the pipeline and Supplementary Information 1.8.5 for the selected hyperparameter values for the elastic net and SVM classifier.

**Fig. 1:**
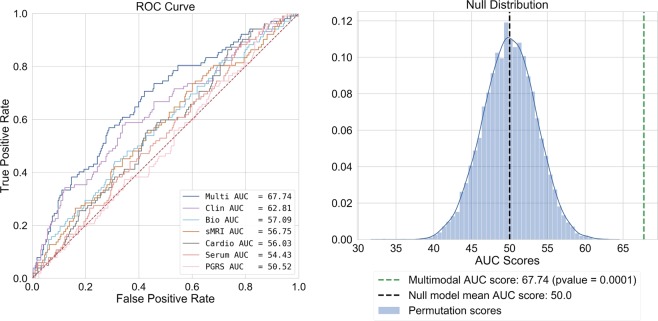
Left: Area under the receiver operator characteristic for all classifiers. Right: Null distribution (blue gaussian distribution) and classifier performance (green dashed line) for our multimodal model after permutation testing *(m* = 10,000)

All steps were completed in an inner cross-validation loop with five repeats of 10-fold cross-validation. This method divides the sample into 10 separate subsets, uses nine for training, and then makes predictions on the final set. To avoid favourable splits in the data, this process is repeated five times, initializing splits uniquely for each repeat. For the testing of our final models, we used 10-fold cross validation in the outer cross validation loop, averaging model performance metrics across test folds. To assess the statistical significance of our final best performing model, we used a permutation test *(m* *=* 10,000) (see Fig. 1).

To assess the predictive capacity of each modality, we trained, clinical, sMRI, cardiovascular, and PGRS only models using the same pipeline as above, as well as a biomarker only model that aggregated together all biomarker modalities. To assess for a significant omnibus effect between models, we used a Kruskal–Wallis *H*-test. Following, we used Mann–Whitney rank tests to assess for post hoc differences between our multi and unimodal models. All *p* values were FDR corrected using the Benjamini and Hochberg method. All ML models were developed using Scikit-learn in Python 3.6.5. All code for analysis is available on request.

### Open sourcing of model

To provide transparency, the use of our model to other research groups, and encourage further external validation of our multimodal model, we uploaded our trained model to the Photon AI online model repository (https://www.photon-ai.com/repo). This repository allows our multimodal model to be downloaded and tested by other research groups.

### Analyses of multimodal predictors

To analyse the direction, magnitude, and significance of the models selected predictors after controlling for covariates, we used a non-penalized implementation of logistic regression in Python’s statsmodels package. In addition, we included the average SVM weight values from the outer 10-fold cross-validation loop from our ML pipeline (Supplementary Information 3.1 and Supplementary Table 2). Further, we conducted ordinary least-squares regression sub-analyses of variables including age, gender, BMI, overall psychotropic medication load, and individual psychotropic medication use on the biomarkers that were selected in the final multimodal model. This was done to elucidate an understanding of variables that may be associated with these biomarkers and help explain their inclusion in the multimodal model. In addition, we conducted an exploratory analysis of patient characteristics for those taking a specific type of medication selected in the multimodal model. This was done to determine: (a) whether this medication proxied for the treatment or prevention of a specific comorbid illness, (b) was taken as a polytheraputic treatment strategy for MDD, or (c) was acting as a proxy for the presence of unmedicated patients that were relapsing at higher rates than the rest of the sample. For these additional analyses and results, see Supplementary Information 3.3–3.5.

## Results

### Multimodal model

Within 2 years of hospitalization for an acute episode of MDD 102 patients (26.8%) were rehospitalized for a depressive episode, while 278 (73.2%) were not. Our best performing model was our multimodal model (test AUC = 67.74). For our multimodal solution, 10 predictors were selected for optimal predictions by the elastic net. Those with a positive association with rehospitalization included the number of previous inpatient depressive episodes, individual CES-D items 5 (Last week I had trouble concentrating) and 3 (In the last week I could not get rid of my mood, although my friends/family tried to cheer me up), Pittsburgh Sleep Quality Index (PSQI) item 7 (During the past month, how often have you had trouble staying awake while driving, eating meals, or engaging in social activity?), the PSQI sleep quality index (global score), taking diazepines, oxazepines, thiazepines and oxepines, and right hippocampal volume. Predictors with a negative association included cholesterol (mmol/l), taking thyroid medications: yes/no, and how often do you drink alcohol? (At most once a week/more than once a week). See Supplementary Figs. 1–3.

Our final multimodal model provided an increase in prognostic certainty of 23.21% (prognostic summary index = (PPV+NPV)–100). Further, a patient classified as being re-hospitalized by our model was 83% more likely to be rehospitalized than a patient who was not (${\mathrm{positive}}\;{\mathrm{likelihood}}\;{\mathrm{ratio}} = \frac{{\mathrm {sensitivity}}}{{1 - \mathrm {specificity}}}$). Finally, our multimodal classifier was statistically significant after permutation testing (*p* *=* 9.99^−e05^) (Fig. 1, Table 3). For individual modality models, see Supplementary Information 3.2.

**Table 3:** Performance metrics for all classifiers

	Train	Test	SVM results										
	AUC	AUC	F1	BAC	Acc	Sens	Spec	PPV	NPV	PSI	PLR	NLR	DOR
Multi	78.86 (2.81)	67.74 (13.86)	67.15	63.05	65.72	57.45	68.65	41.64	81.57	23.21	1.83	0.62	2.96
Clinical	73.59 (1.64)	62.81 (11.14)	64.30	60.10	62.62	54.73	65.44	37.04	79.79	16.83	1.58	0.69	2.29
Bio	63.12 (1.04)	57.09 (11.47)	55.80	51.47	53.64	46.90	56.03	27.44	74.78	2.22	1.07	0.95	1.13
sMRI	64.53 (1.74)	56.75 (10.46)	56.94	52.03	55.31	45.00	59.06	29.16	74.96	4.12	1.10	0.93	1.18
Cardio	61.44 (1.48)	56.03 (13.12)	56.15	54.22	53.65	55.55	52.90	29.62	77.18	6.80	1.18	0.84	1.40
Serum	60.70 (0.75)	54.43 (8.31)	51.81	50.00	63.97	20.00	80.00	5.41	58.57	−36.02	1.00	1.00	1.00
PGRS	59.72 (1.26)	50.52 (13.62)	53.32	50.55	50.85	49.91	51.18	27.88	73.30	1.18	1.02	0.98	1.04

All classifiers used a Linear Support Vector Machine with Platt scaling, only predictor modalities varied across models. Mean (SD) scores from the outer 10-fold cross-validation loops are presented. Model abbreviations: *Multi* our multimodal model (all biomarker modalities, clinical, and demographic variables), *Clinical* clinical and demographic predictors only, *Bio* model with all biomarker modalities (no clinical or demographic data), *sMRI* structural imaging predictor model only, *Cardio* electrocardiography and bioelectrical impedance analysis predictor model only, *Serum* blood biomarkers only, *PGRS* PGRS model only. Metric abbreviations: *AUC* area-under-the-curve, *F1* Harmonic mean of Sens + Spec, *BAC* balanced accuracy, *Acc* accuracy, *Sens* sensitivity, *Spec* specificity, *PPV* positive predicted value, *NPV* negative predicted value, *PSI* prognostic summary index, *PLR* positive likelihood ratio, *NLR* negative likelihood ratio, *DOR* diagnostic odds ratio

## Discussion

The current study is the first of its kind to investigate the role of multiple predictor modalities for MDD rehospitalization trajectory modelling. Overall, our multimodal model provided a 23.21% increase in prognostic certainty for patient rehospitalization classification while providing an isolated subset of multimodal predictors for analysis. Furthermore, our multimodal model was statistically significant after permutation testing.

Of clinical importance is the positive (PPV) and negative predicted values (NPV) of our multimodal model and how they can be used to inform clinical decision making. As PPV was low (PPV = 41.64), using this model to confirm clinician suspicion of rehospitalization is not supported. On the contrary, NPV was modestly high (NPV = 81.57). Suggesting that the model could be used to confirm a clinician’s suspicion of low rehospitalization risk, potentially identifying the patient as suitable for less assertive follow-up.

As few studies are yet to consider a diverse range of biomarker modalities for rehospitalization trajectory modelling, the discriminative ability of the included modalities, as well as their interaction with clinical markers is of interest. First, our best performing model was multimodal, including a range of clinical, blood-biomarker, and structural imaging predictors. A recent work by Koutsouleris et al.^45^ also demonstrated the superiority of multimodal models, finding that a combination of clinical and neuroimaging markers was most predictive of functional outcomes in a cohort of patients at risk for psychosis with recent onset depression. Our findings build on this work as well as provide preliminary evidence for the discriminative ability of blood biomarkers on a well-defined and clinically meaningful dichotomous endpoint.

Given that clinical predictors have consistently demonstrated their discriminative ability in multivariate pattern recognition studies^12,14,46^, it is unsurprising that 8 of 10 predictors in our multimodal solution were clinical. On the contrary, PGRS scores were of no use for rehospitalization classification (test AUC = 50.5) showing the least discriminative ability of all the modelled modalities. Even with lower *p* value thresholds (*p* = 0.05 and 0.01), no changes were seen in our multimodal model. In addition, unimodal PGRS discrimination was still low at these thresholds (AUC = 54.83 and 54.17 respectively, Supplementary Information 3.2). PGRS scores have received strong interest in psychiatric research, hoping that the aggregation of multiple single-nucleotide polymorphisms may illuminate genetic differences in psychiatric traits as well as parse the heterogeneity of outcomes such as medication response^47,48^. Significant associations with outcomes of interest have been consistently demonstrated, however, commonly explain less than 1–2% of variance, limiting their current clinical use. Given that PGRS scores could not classify rehospitalization any better than chance in the current work, the discovery of more variants as well as non-linear modelling techniques may improve their clinical utility in future works^49^. All other biological modalities performed better, yet still lacked clinically meaningful discriminative ability when modelled without clinical predictors. Given these findings and those of Koutsouleris et al.^45^, it appears that biomarkers may be of prognostic use but likely perform best when modelled with clinical predictors also.

Regarding the selection of diazepines, oxazepines, thiazepines, and oxepines and their positive association with rehospitalization, it is possible that patients on this class of drugs at baseline had a more severe form of illness requiring augmentation with antipsychotic medications, placing them at a greater risk of rehospitalization. In addition, thyroid medication use was negatively associated with rehospitalization. Besides a small handful of patients, those with a past diagnosis of hyper/hypothyroidism who were not currently taking thyroid medications had t3, t4, and TSH levels within healthy reference ranges (Supplementary Information 3.3), suggesting that it was not the presence of unmedicated patients with thyroid disorders relapsing at high rates that were responsible for thyroid medication use and its negative association with rehospitalization. Considering that nearly all patients were taking antidepressant medications at their baseline assessment, it is possible that a synergistic prophylactic relationship between thyroid and antidepressant medication use may exist. Such a polytheraputic relationship has been robustly demonstrated in the multisite sequenced alternatives to relieve depression trial (STAR*D)^29^. However, prophylactic effects against rehospitalization should be explored in future works.

Of interest, right hippocampal volume showed a positive association with rehospitalization. This was surprising, given the well-established effect of smaller hippocampal volumes in MDD patients compared to healthy controls^6^. To better understand this observation, we conducted a range of sub-analysis (Supplementary Information 3.4). In accordance with previous works^50,51^, we showed that those taking any form of antipsychotic at baseline had larger right hippocampal volumes than those who were not, albeit, this effect was not significant after controlling for covariates. In addition, we showed that there was a significant gender/diazepine, oxazepine, thiazepine, and oxepine use interaction effect on right hippocampal volumes, with women currently taking medications from this class having significantly larger right hippocampal volumes than those who were not. Forty-four percent of these women ended up being rehospitalized between baseline and their 2-year follow-up assessment, compared to only 22% of women who were not. While we had binary usage data for medication, dosage data were not available. Dosage effects were likely prevalent, but not quantifiable in the current work. Overall, it is plausible that changes in right hippocampal volume proxied for a complex gender/medication/dosage-specific aetiology not fully represented in our clinical data.

The first limitation of the current work is the models’ scope. As our model was trained on a middle-aged European cohort, it is plausible that some of the selected predictors in our pipeline were unique to this demographic. Predictors such as the number of previous inpatient episodes will likely be larger on an older cohort, offering more discriminative ability. Therefore, the clinical utility of the model needs to be considered within this scope. To overcome this limitation, we have provided our trained model online through the Photon AI model repository.

Further limitations include the reporting of nominal significance in our logistic regression model (Supplementary Information 3.1). It is important to consider that the nominally significant effects that were found in our non-penalized logistic regression model did not survive FDR corrections (Supplementary Table 2). However, our primary interest was the emergent multivariate pattern that demonstrated statistically significant class separation between cases and controls at *p* *=* 9.99^−e05^. Given the importance and statistical significance of this aggregated multivariate pattern, we deemed it necessary to conduct an exploratory analysis to illuminate the contribution of each individual predictor after rigorously controlling for known covariates age, gender, smoker status, BMI, and intracranial volume. Given this, we believe the nominal significance of predictors in our logistic regression model to be of use for hypothesis generation in future works.

Regarding clinical use, while our multimodal model was statistically significant after permutation testing, its balanced accuracy was relatively low (BA = 63.05). Whether this level of performance is sufficient for clinical use is unknown; however, this BA is similar to that attained in a previous work that is now deployed clinically (BA = 59.60–64.6)^14^. Further, we argue that it is not the absolute accuracy of a model that should dictate whether it is of clinical use, but whether or not it outperforms current clinical best practice. For example, as rehospitalization risk is not formally and routinely quantified by clinicians to inform their clinical decision making, we would not expect clinician prognostication of rehospitalization to be any better than chance. Therefore, even a modestly performing model would theoretically confer clinical advantage when deployed at scale. In addition, such a model would be free of well-documented decision-making biases that are known to affect clinician prognostication^52^ (e.g., anchoring effects), potentially providing further clinical benefit under high workloads commonly seen in inpatient care. Nonetheless, for these questions to be answered, future works that benchmark clinicians’ prognostic abilities are needed before such incremental utility can be quantified.

Finally, it is important to note that while our multimodal model provided the highest degree of class separation, after testing for post hoc differences between all models and FDR correcting *p* values, our multimodal model did not significantly outperform our model containing only clinical predictors (*p* *=* 0.15) (Supplementary Information 3.2). Future works of increased size may be required to elucidate a statistically significant difference given the small predictive discrepancy between the multimodal and clinical model. On the contrary, clinical data of sufficient depth (for example, that captures the previously discussed gender/medication/dosage effects and their association with right hippocampal volumes) may even mitigate the contribution of biomarkers in future works. If so, these models will bestow both ease of use and economic advantage, rendering the inclusion of costly and harder to attain biomarkers ineffectual. To answer these questions a greater dearth of clinical data, discovery samples of increased size, as well as validation samples that are phenotypically and geographically diverse will be required.

In conclusion, the presented findings suggest that the combination of ML techniques with multimodal clinical and biomarker data may lead to an increase in prognostic certainty compared to chance level. Continued research is required, but ML may be of use to derive models for clinicians to make personalized predictions regarding rehospitalization risk as well as better inform prophylactic treatment strategies.

## Supplementary information

Supplementary Information
