# Supplementary material for: Predicting rehospitalization within 2 years of initial patient admission for a major depressive episode: a multimodal machine learning approach
Source: Transl Psychiatry. 2019 Nov 11;9:285. doi: 10.1038/s41398-019-0615-2 (PMC6848135; doi:10.1038/s41398-019-0615-2)
Supplement: Supplementary file 1 — Supplementary Information [file 41398_2019_615_MOESM1_ESM.docx]

**Supplementary Information**

**Supplementary Information SI1:**

- 1. **Recruitment of participants and ethics statement**

Cohort 1 consisted of 1016 patients, who suffered from an episode of depression at the time of recruitment. Recruitment took place at the Institute of Epidemiology and Social Medicine at the University of Münster, Germany. The recruitment of outpatients was limited to those who had been hospitalized due to depression at least once during the 12 months period prior to inclusion into the study. Inclusion criteria were age (≥35 and < 66 years) and current in- or outpatient treatment due to acute depression. Exclusion criteria were compulsory admission, comorbid dementia, and comorbid drug abuse (including alcohol). Potential participants were ascertained by trained and certified study psychologists, and eligible patients were invited to participate in the BiDirect-Baseline assesment^1^. Appointments were scheduled via telephone or email. All MDD patients included in the present study suffered from an episode of major depression at the time of recruitment and were either currently hospitalized or had been hospitalized due to depression at least once during the 12-month period prior to inclusion into the study according to hospital service records. Further information on hospitalization was obtained via the total number of inpatient depressive episodes. MDD diagnosis was confirmed in all MDD patients with a structured diagnostic interview administered by trained and certified study psychologists. To assess baseline levels of depressive symptoms the Hamilton Rating Scale for Depression (HAM-D) and the Centre for Epidemiologic Studies Depression Scale (CES-D) were administered (appendix methods 1 and 2). The study was approved by the ethics committee of the University of Münster and the Westphalian Chamber of Physicians. All participants provided written informed consent. Further details on rationale, design and recruitment procedures of the BiDirect study have been extensively described in previous publications^11^.

- 1. **Diagnosis of depression**

For diagnostic purposes, all participants received selected modules (i.e. modules A, A’, B, D, and O) of the M.I.N.I. International Neuropsychiatric Interview (German version 5.0.0)^2^, which assessed whether a participant exhibited acute (first or recurrent) major depression with or without melancholic features, acute dysthymia, acute/ former manic/hypomanic episodes, or acute generalized anxiety disorder. Further, for all patients from cohort 1 and for those participants from cohorts 2 and 3 who had received a M.I.N.I. diagnosis of acute major depression, it was clarified whether atypical depression features were currently present by means of six selected items of the Inventory of Depressive Symptomatology (IDS)^3^ (items 8, 12, 14, 27, 29, and 30). In addition, all patients with depression were assessed using the 17 items version of the Hamilton Depression Rating Scale (HAM-D-17)^4^ and the 14 items version of the Hamilton Anxiety Rating Scale (HAM-A-14)^5^.

- 1. **Cognitive function module and self-report predictors**

To measure cognitive function, we included time scores from the trail making test (parts A and B), immediate recall of emotional words from a 12-word passage of positive, neutral, and negative terms, verbal production of different animal names in a one-minute interval, the Purdue Pegboard test, and an 11-point patient self-evaluated attention score. In addition, several self-report questionnaires were available including the Pittsburgh Sleep Quality Index (PSQI), the Pain Sensitivity Questionnaire (PSQ), the Childhood Trauma Screener (CTS) and the EQ-5D, which assesses perceived health-related quality of life. See supplementary table 1 below for all included predictors.

- 1. **Structural MRI procedure**

All structural T1 images were automatically preprocessed using FreeSurfer (Version 5.3) with default parameters. Cortical thickness measures were based on the Desikan–Killiany atlas. Segmentation quality was assessed by visual inspection and statistically evaluated for outliers following a standardized protocol provided by the ENIGMA consortium (<http://enigma.ini.usc.edu/protocols/imaging-protocols)>. T1 structural images were acquired at a single 3.0 T MRI scanner (Intera with Achieva update; Philips Medical Systems, Best, The Netherlands) using a three-dimensional fast gradient echo sequence with a repetition time of 7.26 milliseconds, echo time=3.56 milliseconds, flip angle=9°, two signal averages, inversion prepulse every 404 milliseconds, acquired over a field of view of 256 x 256 mm, phase encoding in AP and RL direction, reconstructed to voxels of 1 mm *x* 1 mm *x* 1 mm.

**1.5 Serum biomarker analysis**

Blood was collected from all patients during examination and serum samples were prepared within two hours after collection and subsequently stored at -80°C in the local biobank until analysis. All laboratory analyses were performed at the Institute of Clinical Chemistry and Laboratory Medicine at the University Medicine Greifswald according to the instructions of the manufacturer using the Dimension Vista® 1500 System (Siemens Healthineers, Eschborn, Germany). Serum samples were stored at -80°C in the Integrated Research Biobank Greifswald after the shipment and until the analysis.

**1.6 Genetic Markers**

The genotyping for the BiDirect cohort was done for 2129 samples using the Infinium PsychArray-24 Kit from Illumina (<https://www.illumina.com/products/by-type/microarray-kits/infinium-psycharray.html>). The genotype data that passed the above QC criteria (n=2115) were imputed in the Michigan server^6^ https://imputationserver.sph.umich.edu using the 1000 Genome Phase 3 version 5, following the ENIGMA protocol (ENIGMA 1KGP_p3v5 Cookbook_20170713.pdf). The genotype data for PGRS scores used were extracted from the imputed dosage using PLINK^7^. See PGRS methods below for more information.

**1.7 Electrocardiogram procedure**

We non-invasively determined vascular status through the measurement of a standard 3-channel electrocardiogram (ECG) using extremity leads, brachial systolic and diastolic blood pressure by oscillometry, the ankle brachial index by photoplethysmography, pulse wave velocity, the augmentation index (Vascular Explorer, enverdis GmbH), and the intima-media-thickness of the far wall of the carotid arteries by ultrasound (Acuson X300, Siemens).

**1.8 Machine learning pipeline architecture**

A Scikit learn pipeline acts as a composite estimator allowing for the chaining of multiple estimators into one. Pipeline architecture affords several advantages. Firstly, parameters across multiple estimators can be tuned in unison, increasing the efficiency of the hyperparameter optimization process. Second, and most importantly, pipelines help avoid leakage from test data into a trained model in the cross-validation process. This is achieved by ensuring that the same samples are used to train the transformers and predictors^8,9^. Within our pipeline we conducted imputation, standardization, feature selection, and hyperparameter optimization.

**1.8.1 Imputation**

Imputation was completed using multivariate imputation by chained equations (MICE). Many multiple imputation techniques assume a large joint model for all variables such as a joint normal distribution. In datasets with *n* variables of different types (continuous, categorical etc), this assumption is not appropriate. MICE is an alternative approach, whereby regression models are run where each variable with missing data is modelled conditional upon the other variables in the data. This approach affords the ability to model each variable according to its own distribution, for example, binary variables are modeled with logistic regression whilst continuous variables are modelled with linear regressions^10^.

**1.8.2 Standardization**

Standardization, also known as zero mean, unit variance ($x^{'}= \frac{x-\bar{x}}{\sigma}$ ) is a common requirement for many machine learning models. Certain models such as radial basis function support vector machines and the l1 and l2 regularizes of linear models (as used in our elastic net feature selection) may perform poorly if the predictors are not centered around zero with variance in the same order. If one predictor has variance that is disproportionately larger than others, it may exert unduly influence over the objective function and make the model unable to learn from other predictors as expected^11^. For this reason, all predictors were standardized within our pipeline.

**1.8.3 Predictor selection**

For predictor selection, we used the elastic net, a form of penalized regression that combines the l1 and l2 regularizes from lasso and ridge regression. This combination allows the learning of a sparse model where some highly correlated coefficients are shrunken to zero as is done in lasso, whilst lesser correlated coefficients are shrunken but non-zeroed^12^. This approach is ideal when a large degree of multicollinearity exists between coefficients, as is common in psychiatric datasets.

**1.8.4 Estimators**

When dealing with small to moderate samples, low complexity (high bias) models tend to provide better fits to the training data. When sample size is constrained, only a small number of hypotheses that map our $x$ matrix to our $y$ vector can be learnt^13-15^. For this reason, we focused on a low complexity linear model, a linear support vector machine. For information on model formulation, see the SVC classification section of the Scikit learn documentation.

**1.8.5 Parameter tuning**

As the overall hyperparameter space was small, we conducted an exhaustive grid search to tune the SVM C and l1 ratio hyperparameters. A grid search allows us to search all specified hyperparameters (parameters not directly learnt within a model) that maximize our cross-validation score according to a specified criterion. In our case, our criterion was the maximization of area under the curve on the receiver operator characteristic. See the [Scikit](http://scikit-learn.org/stable/modules/grid_search.html) learn grid search documentation for more information. For the elastic net, the final hyperparameter values were alpha = 0.3, l1_ratio = 0.1, eps = 0.001, max_iter = -1, tol = 0.0001). For the SVM, the final hyperparameters were C = 0.001, kernel = linear, class_weight = balanced, probability = True).

**1.8.6 Cross Validation**

Cross-validation was conducted using 5 repeats of 10-fold cross-validation. Research suggests that 10-fold cross-validation may better balance the bias-variance trade-off be compared to the more expensive leave-one-out cross-validation. In addition, repeated runs are suggested to avoid favorable splits in the data that may lead to overly optimistic accuracy estimates. Alternatively, bootstrap methods tend to have low variance but extremely high bias on certain datasets. Thus, were avoided in the current analysis. For detailed information, see the work of [Kohavi](http://robotics.stanford.edu/~ronnyk/accEst.pdf)^16^.

**1.8.7 Permutation Testing**

To test whether a model’s classification score is statistically significant, a technique called permutation testing is used. Permutation tests randomly shuffle the class labels and then attempt to make a prediction with the given predictor space. This process is repeated *n* times until a null distribution is attained. Model performance is then compared to that of the null distribution.

**References**

1. Teismann H, Wersching H, Nagel M, et al. Establishing the bidirectional relationship between depression and subclinical arteriosclerosis - rationale, design, and characteristics of the BiDirect Study. *Bmc Psychiatry.* 2014;14.

2. Ackenheil M, Stotz-Ingenlath G, Dietz-Bauer R, Vossen A. MINI mini international neuropsychiatric interview, German version 5.0. 0 DSM IV. *Psychiatrische Universitätsklinik München, Germany.* 1999.

3. Rush AJ, Giles DE, Schlesser MA, Fulton CL, Weissenburger J, Burns C. The inventory for depressive symptomatology (IDS): preliminary findings. *Psychiat Res.* 1986;18(1):65-87.

4. Hamilton M. A rating scale for depression. *J Neurol Neurosurg Psychiatry.* 1960;23:56-62.

5. Hamilton M. The assessment of anxiety states by rating. *Br J Med Psychol.* 1959;32(1):50-55.

6. Das S, Forer L, Schonherr S, et al. Next-generation genotype imputation service and methods. *Nat Genet.* 2016;48(10):1284-1287.

7. Purcell S, Neale B, Todd-Brown K, et al. PLINK: a tool set for whole-genome association and population-based linkage analyses. *Am J Hum Genet.* 2007;81(3):559-575.

8. Kaufman S, Rosset S, Perlich C, Stitelman O. Leakage in Data Mining: Formulation, Detection, and Avoidance. *Acm T Knowl Discov D.* 2012;6(4).

9. Pedregosa F, Varoquaux G, Gramfort A, et al. Scikit-learn: Machine Learning in Python. *J Mach Learn Res.* 2011;12:2825-2830.

10. Azur MJ, Stuart EA, Frangakis C, Leaf PJ. Multiple imputation by chained equations: what is it and how does it work? *Int J Meth Psych Res.* 2011;20(1):40-49.

11. Géron A. *Hands-on machine learning with Scikit-Learn and TensorFlow: concepts, tools, and techniques to build intelligent systems.* " O'Reilly Media, Inc."; 2017.

12. Zou H, Hastie T. Regularization and variable selection via the elastic net. *Journal of the Royal Statistical Society: Series B (Statistical Methodology).* 2005;67(2):301-320.

13. Beleites C, Neugebauer U, Bocklitz T, Krafft C, Popp J. Sample size planning for classification models. *Anal Chim Acta.* 2013;760:25-33.

14. Guo Y, Graber A, McBurney RN, Balasubramanian R. Sample size and statistical power considerations in high-dimensionality data settings: a comparative study of classification algorithms. *Bmc Bioinformatics.* 2010;11.

15. Raudys SJ, Jain AK. Small Sample-Size Effects in Statistical Pattern-Recognition - Recommendations for Practitioners. *Ieee T Pattern Anal.* 1991;13(3):252-264.

16. Kohavi R. A study of cross-validation and bootstrap for accuracy estimation and model selection. Paper presented at: Ijcai1995.

17. Ojala M, Garriga GC. Permutation Tests for Studying Classifier Performance. *J Mach Learn Res.* 2010;11:1833-1863.

**Supplementary Information SI2:**

The genetic profile scores (GPSs) were computed for 33 traits including the major cardiometabolic and psychiatric disorders, metabolic syndrome, the big five personality traits, educational attainment, smoking behavior and childhood behavioral problems.

**2.1 Genotyping and quality control**

For each sample, we implemented quality control (QC) procedures for the genotype data using PLINK^1^ and samples with low genotype rates <95%, sex inconsistencies (X-chromosome heterozygosity), and genetically related individuals were excluded. We also excluded SNPs that had poor genotyping rate <95%, an ambiguity (A/T and C/G SNPs), a minor allele frequency (MAF) < 0.01 or that showed deviation from Hardy-Weinberg Equilibrium (p<10^-6^).

**2.2 Imputations**

The genotype data that passed the above QC criteria were imputed in the Michigan server^2^ <https://imputationserver.sph.umich.edu> separately for each study samples using the Haplotype Reference Consortium (HRC) reference panel^3^.

After excluding the low-frequency SNPs (MAF<0.1); low-quality variants (imputation INFO < 0.9; and indels, the imputed dosages were converted to best guess genotypes with PLINK^1^. The subsequent genetic profile score analyses were performed using the best guess genotypes.

**2.3 Discovery GWAS data**

The genetic profile scores were calculated using the approach previously described by the International Schizophrenia Consortium^4^. This approach requires a discovery and target datasets. The discovery data which refers to the summary data, i.e. effect sizes (beta, a log of odds ratio or log of hazard ratio) in our study were obtained from previous GWASs (see below). The different disorders and phenotypes included were MDD ^5^, bipolar disorder ^6^, schizophrenia^7^, autism spectrum disorders, anxiety disorders ^8^, anorexia nervosa^9^, Alzheimer's disease^10^, type 2 diabetes ^11^, coronary artery disease ^12^, myocardial infarction^12^, lipids level ^13^, body mass index^14^, waist to hip ratio ^15^, glycemic phenotypes (fasting glucose^16^ and fasting insulin^16^, indices of beta-cell function-HOMA-B and insulin resistance-HOMA-IR ^17^, fasting proinsulin^18^, and hemoglobin A₁(C) ^19^, big Five personality traits(extraversion, openness to experience, agreeableness, conscientiousness and neuroticism) ^20,21^, subjective wellbeing ^21^, depressive symptoms ^21^, loneliness ^22^, educational attainment ^23^, smoking behavior^24^, and Childhood behavioral problems^25^.

**2.4 Target sample:** **Study Samples**

Genetic data from three German cohorts were used to compute the genetic profile scores: FOR_2107 (n=922), MS_Imaging_MDD_BD_controls_joint_sample (n=1084) and Marburg_PsychChip (n=374).

**2.5 Genetic profile scoring**

We computed GPS for each of the 33 target traits using the HRC imputed data and GWAS summary statistics of the respective GWASs, separately for the three samples. The GPSs were calculated within a P-value threshold (P_T_) as the sum of reference SNP alleles multiplied by the effect size (β- coefficient or log (OR) or log (HR)) weighted by the sum of effect sizes derived from the GWAS summary statistics. The major histocompatibility complex region was excluded from the GPS calculation because of its complex linkage disequilibrium structure. Quality-controlled SNPs were clumped for linkage disequilibrium based on P-value informed clumping using r2 = 0.1 within a 250-kb window to create a SNP-set in linkage equilibrium using PLINK software run on Linux; *plink–clump-p1 1 –clump-p2 1 –clump-r2 0.1 –clump-kb 250*). The genetic profile scores were computed at different P-value thresholds in the three samples (<5x10^-8^, <1x10^-6^, <1x10^-5^, <1x10^-4^, <1x10^-3^, <0.01, <0.05, <0.1, <0.2, <0.3, <0.4, <0.5, <0.6, <0.7, <0.8, <0.9, <1)

**2.6 List of traits for which genetic profile score was calculated and references for the summary data**

**2.6.1 Cardiometabolic diseases**

- Coronary artery disease-cad_additive_model_2015^12^
- Type 2 diabetes-diagram_mega_type2DM_meta^11^
- Myocardial infarction-mi_additive_model_2015^12^

**2.6.2 Measures of Obesity**

- Body mass index-Obesity_all^14^,
- Waist to hip ratio-GIANT_2015_WHR_COMBINED_AllAncestries ^15^

**2.6.3 Lipids level ^13^**

- jointGwasMc_TG
- jointGwasMc_TC
- jointGwasMc_LDL
- jointGwasMc_HDL

**2.6.4 Glucose and insulin traits**

- Fasting proinsulin: MAGIC_proinsulin^18^
- MAGIC_Manning_et_al_lnFastingInsulin_MainEffect_Oct112012^16^
- MAGIC_Manning_et_al_FastingGlucose_MainEffect_Oct112012^16^
- MAGIC_ln_HOMA_IR^17^
- MAGIC_ln_HOMA_B^17^
- Hemoglobin A₁(C)- MAGIC_HbA1C^19^

**2.6.5 Psychiatric disorders**

- Anxiety disorders- Anxiety_case_control_2016 ^8^
- Bipolar disorder-pgc_bip_full_2012^6^
- MDD- pgc_mdd_full_2012 ^5^ and MDD23andme^26^
- Schizophrenia-PGC_SCZ2_2014^7^
- Alzheimer's disease- IGAP_Alzheimer^10^
- Anorexia nervosa: PGC_anorexia_snp_all_13May2016^9^
- PGC_ASD_AUD_5Mar2015: *Autism Spectrum Disorder Working Group of the Psychiatry Genomics Consortium. Dataset: PGC-ASD summary statistics from a meta-analysis of 5,305 ASD-diagnosed cases and 5,305 pseudocontrols of European descent (March 2015). (available at: http://www.med.unc.edu/pgc/results-anddownloads)*

**2.6.6 Psychological traits**

- Subjective wellbeing-SWB_Full ^21^
- Depressive symptoms-DS_Full ^21^
- Loneliness-in continuous and dichotomous scales ^22^

**2.6.7 Big Five personality traits**

- GPC_1_NEO_AGREEABLENESS^20^
- GPC_1_NEO_CONSCIENTIOUSNESS^20^
- GPC_1_NEO_EXTRAVERSION^20^
- GPC_1_NEO_OPENNESS^20^ and
- NEUROTICISM_Full^21^

**2.6.8 Smoking behavior**^24^

- Cigarates_per_day_continuous
- Cigarates_Ever_never_Categorical
- Cigarates_Current_former_Categorical
- Cigarates_Ageofonset_continuous

**2.6.9 Educational attainment and Childhood behavioral problems**

- EduYears_2016^23^
- Preschool_Internalizing_Problem^25^

**PGRS references**

1 Purcell, S. *et al.* PLINK: a tool set for whole-genome association and population-based linkage analyses. *Am J Hum Genet* **81**, 559-575, doi:10.1086/519795 (2007).

2 Das, S. *et al.* Next-generation genotype imputation service and methods. *Nat Genet* **48**, 1284-1287, doi:10.1038/ng.3656

http://www.nature.com/ng/journal/v48/n10/abs/ng.3656.html#supplementary-information (2016).

3 McCarthy, S. *et al.* A reference panel of 64,976 haplotypes for genotype imputation. *Nat Genet* **48**, 1279-1283, doi:10.1038/ng.3643

<http://www.nature.com/ng/journal/v48/n10/abs/ng.3643.html#supplementary-information> (2016).

4 Purcell, S. M. *et al.* Common polygenic variation contributes to risk of schizophrenia and bipolar disorder. *Nature* **460**, 748-752, doi:10.1038/nature08185 (2009).

5 Ripke, S. *et al.* A mega-analysis of genome-wide association studies for major depressive disorder. *Mol Psychiatry* **18**, 497-511, doi:10.1038/mp.2012.21 (2013).

6 Large-scale genome-wide association analysis of bipolar disorder identifies a new susceptibility locus near ODZ4. *Nat Genet* **43**, 977-983, doi:10.1038/ng.943 (2011).

7 Biological insights from 108 schizophrenia-associated genetic loci. *Nature* **511**, 421-427, doi:10.1038/nature13595 (2014).

8 Otowa, T. *et al.* Meta-analysis of genome-wide association studies of anxiety disorders. *Mol Psychiatry*, doi:10.1038/mp.2015.197 (2016).

9 Boraska, V. *et al.* A genome-wide association study of anorexia nervosa. *Mol Psychiatry* **19**, 1085-1094, doi:10.1038/mp.2013.187 (2014).

10 Lambert, J. C. *et al.* Meta-analysis of 74,046 individuals identifies 11 new susceptibility loci for Alzheimer's disease. *Nat Genet* **45**, 1452-1458, doi:10.1038/ng.2802 (2013).

11 Mahajan, A. *et al.* Genome-wide trans-ancestry meta-analysis provides insight into the genetic architecture of type 2 diabetes susceptibility. *Nat Genet* **46**, 234-244, doi:10.1038/ng.2897 (2014).

12 Nikpay, M. *et al.* A comprehensive 1,000 Genomes-based genome-wide association meta-analysis of coronary artery disease. *Nat Genet* **47**, 1121-1130, doi:10.1038/ng.3396 (2015).

13 Global Lipids Genetics, C. Discovery and refinement of loci associated with lipid levels. *Nat Genet* **45**, 1274-1283, doi:10.1038/ng.2797

http://www.nature.com/ng/journal/v45/n11/abs/ng.2797.html#supplementary-information (2013).

14 Locke, A. E. *et al.* Genetic studies of body mass index yield new insights for obesity biology. *Nature* **518**, 197-206, doi:10.1038/nature14177 (2015).

15 Shungin, D. *et al.* New genetic loci link adipose and insulin biology to body fat distribution. *Nature* **518**, 187-196, doi:10.1038/nature14132 (2015).

16 Manning, A. K. *et al.* A genome-wide approach accounting for body mass index identifies genetic variants influencing fasting glycemic traits and insulin resistance. *Nat Genet* **44**, 659-669, doi:<http://www.nature.com/ng/journal/v44/n6/abs/ng.2274.html#supplementary-information> (2012).

17 Dupuis, J. *et al.* New genetic loci implicated in fasting glucose homeostasis and their impact on type 2 diabetes risk. *Nat Genet* **42**, 105-116, doi:10.1038/ng.520 (2010).

18 Strawbridge, R. J. *et al.* Genome-wide association identifies nine common variants associated with fasting proinsulin levels and provides new insights into the pathophysiology of type 2 diabetes. *Diabetes* **60**, 2624-2634, doi:10.2337/db11-0415 (2011).

19 Soranzo, N. *et al.* Common variants at 10 genomic loci influence hemoglobin A(1)(C) levels via glycemic and nonglycemic pathways. *Diabetes* **59**, 3229-3239, doi:10.2337/db10-0502 (2010).

20 de Moor, M. H. *et al.* Meta-analysis of genome-wide association studies for personality. *Mol Psychiatry* **17**, 337-349, doi:10.1038/mp.2010.128 (2012).

21 Okbay, A. *et al.* Genetic variants associated with subjective well-being, depressive symptoms, and neuroticism identified through genome-wide analyses. *Nat Genet* **48**, 624-633, doi:10.1038/ng.3552

http://www.nature.com/ng/journal/v48/n6/abs/ng.3552.html#supplementary-information (2016).

22 Gao, J. *et al.* Genome-Wide Association Study of Loneliness Demonstrates a Role for Common Variation. *Neuropsychopharmacology*, doi:10.1038/npp.2016.197 (2016).

23 Okbay, A. *et al.* Genome-wide association study identifies 74 loci associated with educational attainment. *Nature* **533**, 539-542, doi:10.1038/nature17671

http://www.nature.com/nature/journal/v533/n7604/abs/nature17671.html#supplementary-information (2016).

24 Genome-wide meta-analyses identify multiple loci associated with smoking behavior. *Nat Genet* **42**, 441-447, doi:<http://www.nature.com/ng/journal/v42/n5/suppinfo/ng.571_S1.html> (2010).

25 Benke, K. S. *et al.* A genome-wide association meta-analysis of preschool internalizing problems. *Journal of the American Academy of Child and Adolescent Psychiatry* **53**, 667-676.e667, doi:10.1016/j.jaac.2013.12.028 (2014).

26 Hyde, C. L. *et al.* Identification of 15 genetic loci associated with risk of major depression in individuals of European descent. *Nat Genet* **48**, 1031-1036, doi:10.1038/ng.3623

http://www.nature.com/ng/journal/v48/n9/abs/ng.3623.html#supplementary-information (2016).

**Supplementary Information SI3:**

- 1. **Logistic regression and SVM weights**

To understand the contributions of the 10 selected predictors in our multimodal model, we used logistic regression and controlled for demographic covariates age and gender and general health covariates, smoker status and BMI (of which may have confounding effects on cholesterol and right hippocampal volumes). In addition, we controlled for intracranial volume to adjust for the natural variability of brain size in this analysis. Due to the multicollinearity between variables, we standardised all variables to have a mean of zero and a standard deviation of one. As this analysis is exploratory/hypothesis generating and secondary to the emergent multivariate pattern that was statistically significant in our multimodal model, we only report on variables that attained or were approaching nominal significance in this section (with nominal p-values reported). FDR corrected p-values and all other variables from our multivariate pattern can be found in table 2.

For every 1 standard deviation increase in the number of previous hospital admissions recorded at baseline, patients became 50% more likely to be rehospitalized within the following two-years post assessment [OR: 1.50, 95% CI: 1.11 – 2.03, *p* = 0.009]. In addition, for every 1 standard deviation increase in right hippocampal volume, patients were 72% more likely to be rehospitalized in the following 2 years [OR: 1.72, 95% CI: 1.13 – 2.62, *p* = 0.01]. Of note, the following predictors approached nominal significance and may be worthy of investigation in future works. Those taking thyroid medications at baseline were 59% less likely to be rehospitalized in the following 2 years [OR: 0.41, 95% CI: 0.15 – 1.10, *p* = 0.08], whilst those who had trouble concentrating ‘mostly or all of the time’ on CES-D item 5, were more than 3 times as likely to be rehospitalized in the following two years [OR: 3.12, 95% CI: 0.83 – 11.77, *p* = 0.09]. For all other results, see Table 2.

Finally, whilst the odds ratios and associated p-values from our logistic regression model allow us to interpret the univariate relationships between each predictor and rehospitalization after controlling for clinically relevant covariates, it is not analogous to the SVM weight values for each predictor that were used to the construct the hyperplane in our final multimodal classification model. Therefore, we have also taken the final average weight values for each selected predictor across the outer 10-fold cross-validation loop from our multimodal ML pipeline. In descending order, SVM weights were: CES-D 5 (Last week I had trouble concentrating) = 0.1015, PSQI Sum: PSQI sleep quality index (global score) = 0.0948, Number of inpatient depressive episodes = 0.0925, PSQI 7 (Difficulties staying awake) = 0.0871, CES-D 3 (Could not get rid of my troubling mood) = 0.0847, Take Diazepines, oxazepined, thiazepines and oxepines? = 0.0717, Right hippocampal volume = 0.0560, Total cholesterol = -0.0450, Thyroid therapy? = -0.0511, How often do you drink an alcoholic beverage? = -0.0650.

- 1. **Individual modality models**

We re-trained our classifier with individual data modalities only, including clinical, sMRI, serum markers, cardiovascular markers, PGRS, as well as a combination of all biomarker modalities. The pipeline used was the same as that in our multimodal analysis. However, as the size of the predictor space was small for our individual biological modalities, we used all a-priori selected predictors with no elastic net predictor selection for the sMRI, serum, cardiovascular and PGRS models. Due to the higher number of predictors in our clinical modality and combined biomarker model, a smaller subset was selected with the elastic net as per the multimodal model pipeline. Results for the following models were: clinical modality model (train AUC = 73.59, test AUC = 62.81) our combined biomarker model (train AUC = 63.12, test AUC = 57.09), sMRI model (train AUC = 64.53, test AUC = 56.75), cardiovascular model (train AUC = 61.44, test AUC = 56.03), serum model (train AUC = 60.70, test AUC = 54.43), and PGRS model (train AUC = 59.72, test AUC = 50.52). Significant differences were found between models after correcting for ties (H = 11.42, *p* = 0.04). Following FDR corrections, our multimodal model performed significantly better than our combined biomarker model (*p =* 0.04), serum model (*p* = 0.03), sMRI model (*p* = 0.05), cardio model (*p* = 0.04), and our PGRS model (*p* = 0.03), but not our clinical model (*p* = 0.15). For the unimodal PGRS models with lowered p-value thresholds (0.05 and 0.01), we attained test AUCs of 54.83 and 54.17.

- 1. **Were thyroid medications protective against rehospitalization?**

To investigate the negative association between thyroid medication and rehospitalization, we looked at the proportions of those with and without a past thyroid disorder diagnosis relative to those taking thyroid medications at their baseline assessment. Fifty-eight patients had a past thyroid diagnosis at baseline and were taking thyroid medications at their assessment. In addition, 29 patients had a past diagnosis of a thyroid disorder and were not taking thyroid medications at their baseline assessment. Of these patients, 25 had t3 levels greater than 3.5 mmol/l, whilst four had levels that went down to 3.08 mmol/l. No patients exceeded 5.24 mmol/l. For t4, all 29 patients were in the healthy reference range (9 mmol/l to 25 mmol/l). For TSH, 26 had levels in the healthy range (0.04 mU/L to 4.0 mU/L) whilst 3 dropped as low as 0.02 mU/L. Finally, 5 patients were taking thyroid medications with no diagnosis of a thyroid condition, presumably as a polytheraputic treatment strategy. Besides 1 patient with a TSH of 0.01 mU/L, all thyroid markers for all 5 patients were in their healthy reference ranges.

- 1. **Were right hippocampal volumes effected by medication use?**

Previous studies have consistently demonstrated smaller hippocampal volumes in MDD patients compared to healthy controls^6^. Surprisingly, larger right hippocampal volumes were predictive of rehospitalization in our logistic regression model (Rehospitalized? **Yes**: M = 4141.54, SD = 430.16, **No**: M = 4002.74, SD = 484.74). To elucidate an explanation for this effect, we conducted a range of sub-analysis, controlling for age, gender, and intracranial volume in all analyses.

As nearly all patients were on an antidepressant medication at their baseline inpatient assessment, teasing out neurogenic effects of antidepressants on hippocampal volume was not possible. However, previous studies have shown that the use of antipsychotics may also affect hippocampal volumes^46-48^. Of interest, 40.3% (153/380) patients were taking an antipsychotic medication as augmentation to normal antidepressant therapy. To assess the effect of these medications on right hippocampal volume we used ordinary least squares regression. According to the minimization of the Akaike information criterion^49^, we found a significant gender/medication use interaction for the antipsychotic class diazepines, oxazepines, thiazepines, and oxepines. Medications that fall into this class include Olanzapine, Clozapine, Loxapine, Quetiapine, and Asenapine. Specifically, we found that women on this class of medication had significantly larger right hippocampal volumes (M = 4017.9, SD = 397.03) than women who were not (M = 3846.58, SD = 336.51) (*b* = 270.09, CI = 63.89 – 476.3, *p* = 0.01). Further, 44.7% of women who were on this class of medication at baseline were re-hospitalized between baseline and their 2 year follow up assessment, whilst only 22% of women who were not were rehospitalized within this period. However, the main effect for diazepine, oxazepine, thiazepine, and oxepine use at baseline was negative and non-significant (*b* = -95.39, CI: -248.25 – 57.46, *p* = 0.22). In aggregate, those taking any form of other antipsychotic medication (classes include phenothiazines with piperidine structure, butyrophenone derivatives, thioxanthene derivatives, benzamides, and “other antipsychotics”) had larger hippocampal volumes (M = 4139.13, SD = 475.47) compared to those who were not (M = 4028.48, SD = 473.28), however, this difference was not significant (*b* = 85.58, CI: -55 – 226.18, *p* = 0.23). Finally, we found smaller right hippocampal volumes in women (M = 3875.26, SD = 352.3) compared to men (M = 4288.14, SD = 524.3), however, these differences were not significant after controlling for intracranial volume (*b* = -46.63, CI: -141.35 – 48.1, *p* = 0.33). In addition, we found a significant relationship between age and right hippocampal volume. As age increased, right hippocampal volumes significantly decreased (*b* = -8.5, CI: -13.5 - -3.5, *p* = 0.001).

- 1. **Were cholesterol levels affected by medication use?**

Twenty-eight patients were taking lipid modifying agents at their baseline assessment. To assess the effects of these medications on cholesterol levels we controlled for covariates age, gender, and BMI and ran an ordinary least squares regression onto cholesterol levels (mmol/l). For patients taking lipid modifying agents, we found a statistically significant decrease of 0.80 mmol/l in cholesterol levels compared to those who were not taking these medications (*b* = 0.80, CI: -1.19 - -0.41, *p* = < 0.0001).

**Supplementary Table 1.** Variable table for all considered predictors entered into the multimodal pipeline for selection by the Elastic Net and subsequent SVM training.

| **Stuctural imaging** | **Freesurfer - volume and thickness** |
| --- | --- |
| Lhippo | Left hippocampal volume |
| Rhippo | Right hippocampal volume |
| L_medialorbitofrontal_thickavg | Left medial orbitofrontal thickness average |
| R_medialorbitofrontal_thickavg | Right medial orbitofrontal thickness average |
| L_fusiform_thickavg | Left fusiform thickness average |
| R_fusiform_thickavg | Right fusiform thickness average |
| L_insula_thickavg | Left insula thickness average |
| R_insula_thickavg | Right insula thickness average |
| L_rostralanteriorcingulate_thickavg | Left rostral anterior cingulate thickness average |
| R_rostralanteriorcingulate_thickavg | Right rostral anterior cingulate thickness average |
| L_posteriorcingulate_thickavg | Left posterior cingulate thickness average |
| R_posteriorcingulate_thickavg | Right posterior cingulate thickness average |
| L_middletemporal_thickavg | Left middle temporal thickness average |
| R_inferiortemporal_thickavg | Right inferior temporal thickness average |
| R_caudalanteriorcingulate_thickavg | Right caudal anterior cingulate thickness average |
| **Blood biomarkers** |  |
| andrix | Free androgen index |
| b17o | 17 beta estradiol |
| shbg | Sex hormone binding hormone |
| testosteron | Testosterone |
| cholesterol | Total cholesterol |
| ft3 | Free triiodothyronine (pmol) |
| hdl | High density lipoprotein cholesterol (mmol/l) |
| ft4 | Free thyroxine (pmol) |
| tsh | Thyroid stimulating hormone (mU) |
| hscrp | CRP highly sensitive (mg/l) |
| **Polygenic risk scores** |  |
| anxiety_pgrs_score | p-value threshold p = 0.5 |
| alzheimer_pgrs | p-value threshold p = 0.5 |
| anorexia_pgrs | p-value threshold p = 0.5 |
| asd_pgrs | p-value threshold p = 0.5 |
| pgc_bip_pgrs | p-value threshold p = 0.5 |
| pgc_mdd_pgrs | p-value threshold p = 0.5 |
| pgc_scz_pgrs | p-value threshold p = 0.5 |
| **Cardiovascular health** | **Electrocardiogram and bioelectrical impedence analysis** |
| ekg_hr | Heart rate beats per minute |
| bia_bmi | Calculated body mass index kg / m 2 |
| bia_ecm_bcm_index | ECM BCM Ratio |
| bia_grundumsatz | Basic metabolic rate kcal |
| bia_kfett_k_kg | Corrected body fat in kg |
| bia_koerperwasser | Total body water kg |
| bia_magermasse | Lean body mass kg |
| **Habit change** |  |
| s_lsstair | Changed habits? Use the stairs more often |
| s_lswalk | Changed habits? Do frequent shopping on foot |
| s_lsweight | Changed habits? decrease weight loss |
| s_lsalc | Changed habits? Drink less alcohol |
| s_lssmoke | Changed habits? give up smoking |
| **Demographic** |  |
| sex | Gender |
| sf1 | How would you describe your state of health at the moment? |
| job_f1 | Are you currently employed? |
| szlage | How would you assess your own economic situation at the moment? |
| szeink | What is the total monthly net income of your household? |
| rfdiat | Keep a specific diet or diet. If yes, which ones? |
| sm_f1 | Currently smoke cigarettes pipes or cigars? |
| sz_mde1 | If you have a disability card, please state the severity of your disability in a percentage. |
| minia1 | A1 In the last 2 weeks, you felt depressed or depressed almost every day, almost all day long? |
| minia2 | A2 In the last 2 weeks have you almost always had the feeling that you are tired and that you have lost interest joy in things that usually make you happy? |
| alter1 | Age at first contact? |
| weight | Measured weight kg |
| edu_f1 | Which is your highest school or university degree? |
| edu_f2 | Which is your highest vocational degree? |
| **Clinical symptoms** | **HAM-D, HAM-A, CES-D, IDS, EQ-5D** |
| ph_hamd1 | Depressed mood |
| ph_ids8 | Mood Reaction |
| ph_hamd2 | Work and other activities |
| ph_ids4 | Hypersomnia |
| ph_hamd3 | Sleep Disorders / Difficulties to fall asleep |
| ph_hamd4 | Sleeping disturbances |
| ph_hamd5 | Early awakening |
| ph_hama6 | Sleep disorders |
| ph_hama7 | Depressed mood |
| ph_hama8 | Intellectual skills |
| ph_hamd9 | Genital tract related symptoms |
| ph_hama10 | Urogenital symptoms |
| ph_hamd11 | Somatic symptoms Gastrointestinal |
| ph_ids12 | Appetite Increases |
| ph_ids14 | Weight gain within the last 14 days |
| ph_hamd12 | Weight loss assessment according to the medical history |
| ph_hamd13 | General somatic symptoms |
| ph_ids30 | Lead Lore Physical Energy |
| ph_hamd14 | Guilty |
| ph_hamd15 | Suicidal thoughts |
| ph_hama16 | Anxious mood |
| ph_hamd17 | Anxiety Mental symptoms |
| ph_hama18 | Inner Tension |
| ph_hama19 | Somatic muscle discomfort |
| ph_hama20 | Somatic Sensory Discomfort |
| ph_hama21 | Fear |
| ph_hama22 | Gastrointestinal symptoms |
| ph_hama23 | Vegetative symptoms |
| ph_hama24 | Cardiovascular symptoms |
| ph_hama25 | Respiratory symptoms |
| ph_hamd26 | Anxiety somatic symptoms |
| ph_ids29 | Interpersonal Sensitivity |
| ph_hamd27 | Hypochondria |
| ph_hamd28 | Disease insight |
| ph_hamd29 | Agitation |
| ph_hama30 | Behavior during the interview |
| ph_hamd31 | Slowdown |
| pi_n_epi_inpatient | Number of inpatient episodes? |
| pi_n_episodes | Total number of depressive episodes? |
| pi_n_inpatient | Total number of hospitilizations? |
| pi_age_1episode | Age at first depressive episode |
| pi_dat_prevepi | Month and Year of onset of last episode? |
| pi_dat_1inpatient | Month and year of first hospitalization? |
| pi_curepi_year | Year of onset of current depressive episode |
| hamd_17total | HAM D total score 17 items |
| hamd_17n | HAM D answers to the 17 items |
| hama_14total | HAM A total score 14 items |
| hama_14n | HAM A answers to the 14 items |
| pm_a2 | In the last 2 weeks you have almost always had the feeling that you are tired of everything and that you have lost interest and the joy of things that usually make you happy? |
| pm_lifa7_a | Have you experienced the feeling of depression differently than the feeling of sadness in the death of a loved one? |
| pm_lifa7_b | Did you feel worse in the morning compared to evening? |
| pm_lifa7_c | Wake up almost twice an hour or earlier than usual and then have trouble falling asleep again? |
| pm_lifa7_d | Have you talked slower than usual almost every day, or were you restless and unable to sit still? |
| pm_lifa7_e | Have lost or had increases in your appetite almost every day? Gain or lose weight? |
| pm_lifa7_f | Feelings of excess guilt? |
| pm_lifepis_n | Melancholic symptoms occurred in episode? |
| pm_lif_age | Age of this melancholic episode? |
| pm_d1_a | Have you ever had a time when you felt so overflowing and energetic that it caused trouble? |
| pm_d2_a | Have you ever had a time in which you constantly felt so irritable for several days that you got into verbal or physical strife or yells at someone else? |
| pm_o1_a | In the past 6 months, have you been overly anxious and worried about many different things in daily life, such as your financial situation, your work, family, or your friends? |
| pm_a4 | Episode of major depression current ??? |
| cesd1 | In the last week things have worried me that usually do not worry me |
| cesd2 | I hardly had any appetite during the last week |
| cesd3 | In the last week I could not get rid of my troblesome mood although my friends family tried to cheer me up |
| cesd4 | Last week, I felt as good as others |
| cesd5 | In the last week I had trouble to concentrate |
| cesd6 | In the last week I was depressed |
| cesd7 | In the last week everything was exhausting for me |
| cesd8 | Last week, I thought of the future with hope |
| cesd9 | Last week, I thought my life was a single failure |
| cesd10 | I was often scared in the last week |
| cesd11 | I slept badly last week |
| cesd12 | Last week I was happy |
| cesd13 | In the last week, I've talked less than usual |
| cesd14 | In the last week I felt lonely |
| cesd15 | In the last week, the people were unkind to me |
| cesd16 | In the last week, I enjoyed life |
| cesd17 | I had to cry sometimes in the last week |
| cesd18 | I was sad the last week |
| cesd19 | In the last week I had the feeling that people do not like me |
| cesd20 | In the last week, I could not get going |
| cesdsum | CES D Sum |
| cesd2c | CES D 0 16 points 1 16 points |
| cesd3c | CES D 0 15 points 1 15 and 21 points 2 21 points |
| cesd_dp | CES D depression items 3 6 9 10 14 17 18 Subscale |
| cesd_wb | CES D well being items 4 8 12 16 REVERSED Subscale |
| cesd_so | CES D somatic items 1 2 5 7 11 13 20 Subscale |
| cesd_ip | CES D inter personal items 15 19 Subscale |
| eq5d_2 | EQ 5D 3L Item 2 take care of yourself |
| eq5d_3 | EQ 5D 3L Item 3 general activities |
| eq5d_4 | EQ 5D 3L Item 4 pain physical discomfort |
| eq5d_5 | EQ 5D 3L Item 5 Fear depression |
| eq5d_vas | EQ 5D 3L thermometer today's state of health |
| eq5dscore | EQ 5D Score European VAS value set |
| **Childhood trauma** | **Childhood trauma screener** |
| cts_1 | CTS Childhood Item 1 was loved |
| cts_2 | CTS Childhood Item 2 physical violence in the family |
| cts_3 | CTS Childhood Items 3 Hatred in the family |
| cts_4 | CTS Childhood Item 4 sexually debased |
| cts_5 | CTS Childhood Item 5 taken to doctor if needed |
| ctssum | CTS Sumscore about Items 1 to 5 Inversion of Items 1 and 5 takes into account possible range 5 to 25 |
| **Sleep** | **Pittsburg sleep quality index** |
| psqi_1 | When have you usually gone to bed? |
| psqi_2_ | How long (in minutes) has it taken you to fall asleep each night? |
| psqi_3 | When have you usually gotten up in the morning? |
| psqi_4 | How many hours of actual sleep do you get at night? (This may be different than the number of hours you spend in bed) |
| psqi_5_sum | During the past month, how often have you had trouble sleeping because… (sum of items a – j) |
| psqi_6 | During the past month, how often have you taken medicine (prescribed or “over the counter”) to help you sleep? |
| psqi_7 | During the past month, how often have you had trouble staying awake while driving, eating meals, or engaging in social activity? |
| psqi_8 | During the past month, how much of a problem has it been for you to keep up enthusiasm to get things done? |
| psqi_9 | During the past month, how would you rate your sleep quality overall? |
| psqilaten | PSQI subcomponent Sleep latency |
| psqidurat | PSQI subcomponent sleep duration |
| psqihse | PSQI subcomponent habitual sleep efficiency |
| psqidistb | PSQI subcomponent sleep disturbances |
| psqidaydys | PSQI subcomponent Daytime dysfunction |
| psqi_sum | PSQI sleep quality index global score |
| **Cognition** |  |
| d_ewo_d1n | Memory (~VLMT) for emotional words: 1st pass, number correct |
| d_ewo_d1w01 | Memory (~VLMT) for emotional words: 2nd pass, number correct |
| d_ewo_d1w02 | Memory (~VLMT) for emotional words: 3rd pass, number correct |
| d_flue_r | Word fluidity – Animal words stated in 1 min interval |
| d_peg_li | Pegboard test, left hand, number of correctly set pins |
| d_peg_re | Pegboard test, right hand, number of correctly set pins |
| d_tmt_a | Trail making test, part A, duration |
| d_tmt_b | Trail making test, part B, duration |
| d_self | Attention self-evaluation: 0 - 10 |
| **Diet and alcohol consumption** |  |
| diet_score | Diet Quality Score (range 0 to 30) |
| al_f1 | How often do you drink an alcoholic beverage of beer, spirits, liquor mixes, etc? |
| al_f3_b | How much beer, wine or liquor did you drink yesterday in liters? |
| al_f4_b | How much beer and wine did you drink on the last weekend in liters? |
| al_f5 | How often does it happen that you drink 6 or more alcoholic drinks on one occasion? |
| al_f8 | Have your spouse or other close relatives or friends ever worried or complained about drinking alcohol? |
| basicsum | BASIC alcohol FB sum score points |
| alkohol | average alcohol consumption last week grams |
| **Physical pain** | **Pain sensitivity questionaire** |
| ks_allks | Headache General Headache |
| ks_mig | Headache Migraine |
| psq_mean | Mean Pain Sensitivity Questionnaire includes Items PSQ1 to PSQ7 |
| dx_chschmerz | Chronic Pain Diagnosis? |
| **Medication** |  |
| med_a03 | Drugs for functional gastrointestinal disorder |
| med_a10 | Drugs used in diabetes |
| med_a11 | vitamins |
| med_a12 | Mineral supplements |
| med_c07 | Beta blocking agents |
| med_c08 | Calcium channel blockers |
| med_c09 | Agents acting on the renin angiotensin system |
| med_c10 | Lipid modifying agents |
| med_g03 | Sex hormones and modulators of the genital system |
| med_g04 | Urologicals |
| med_h02 | Corticosteroids for systemic use |
| med_h03 | Thyroid therapy |
| med_l04 | immunosuppressants |
| med_m03 | Muscle relaxants |
| med_n02aa | Natural opium alkaloids |
| med_n02ab | Phenylpiperidines derivatives |
| med_n02ae | Oripavine derivatives |
| med_n02ax | Other opioids |
| med_n02ba | Salicylic acid and derivatives |
| med_n02bb | Pyrazolones |
| med_n02be | Anilides |
| med_n02bg | Other analgetics and antipyretics |
| med_n02cc | Selective serotonin agonists |
| med_n03ae | Benzodiazepine derivatives |
| med_n03af | Carboxamide derivatives |
| med_n03ag | Fatty acid derivatives |
| med_n03ax | Other antiepileptics |
| med_n05ab | Phenothiazines with piperazine structure |
| med_n05ad | Butyrophenone derivatives |
| med_n05af | Thioxanthene derivatives |
| med_n05ah | Diazepines oxazepined thiazepines and oxepines |
| med_n05al | Benzamide |
| med_n05an | Lithium |
| med_n05ax | Other antipsychotics |
| med_n05ba | Benzodiazepine derivatives |
| med_n05cd | Benzodiazepine derivatives |
| med_n05cf | Benzodiazepines related drugs |
| med_n05ch | Melatonin receptor agonists |
| med_n05cm | Other hypnotics and sedatives |
| med_n06aa | Non-selective momoamine reuptake inhibitors |
| med_n06ab | Selective serotonin reuptake inhibitors |
| med_n06af | Monoamine oxidase inhibitors non selective |
| med_n06ax | Other antidepressants |
| med_v06 | General nutrients |
| **Exercise** |  |
| ipaq_f1d | On how many of the past 7 days have you been doing strenuous physical activities such as heavy lifting, digging aerobics or fast biking, number of days |
| ipaq_f2h | How much time did you usually spend on one of these days of strenuous physical activity? hours per day |
| ipaq_f3d | On how many of the past 7 days do you have moderate physical activities? number of days |
| ipaq_f4h | How much time did you usually spend on one of these days with moderate physical activity? hours per day |
| ipaq_f5d | On how many of the past 7 days have you walked at least 10 minutes without interruption? number of days |
| ipaq_f6h | How much time did you usually spend walking on one of these days? hours |
| ipaq_f7h | How much time have you spent sitting over the last 7 days? hours |
| ipaq_total_met | IPAQ total score |
| ipaq_category | IPAQ categorical score 3 levels of physical activity |

**Supplementary Table 2.** Summary of our logistic regression model for the final 10 predictors of rehospitalization after controlling for age, gender, BMI, smoker status, and intracranial volume.

|  | Estimate | Std Error | Statistic | OR | 2.5% CI | 97.5% CI | P Val | FDR P Val |
| --- | --- | --- | --- | --- | --- | --- | --- | --- |
| Intercept | -1.64 | 0.72 | -2.27 | 0.19 | 0.05 | 0.80 | 0.02 | 0.18 |
| CES-D 5 (trouble concentrating?) - Sometimes | 0.67 | 0.60 | 1.11 | 1.96 | 0.60 | 6.40 | 0.27 | 0.60 |
| CES-D 5 (trouble concentrating?) - More often | 0.29 | 0.64 | 0.46 | 1.34 | 0.38 | 4.68 | 0.64 | 0.82 |
| CES-D 5 (trouble concentrating?) - Mostly | 1.14 | 0.68 | 1.68 | 3.12 | 0.83 | 11.77 | 0.09 | 0.37 |
| Diazepines oxazepines thiazepines oxepines - Yes | 0.36 | 0.33 | 1.09 | 1.43 | 0.75 | 2.71 | 0.28 | 0.60 |
| PSQI 7 (Trouble staying awake?) - Less than once a week | 0.18 | 0.58 | 0.32 | 1.20 | 0.39 | 3.71 | 0.75 | 0.83 |
| PSQI 7 (Trouble staying awake?) - One or two times a week | 0.19 | 0.62 | 0.31 | 1.21 | 0.36 | 4.05 | 0.76 | 0.83 |
| PSQI 7 (Trouble staying awake?) - Three or more times a week | 0.44 | 0.39 | 1.12 | 1.56 | 0.72 | 3.37 | 0.26 | 0.60 |
| CES-D 3 (could not get rid of mood) - Sometimes (1-2 days) | -0.73 | 0.43 | -1.71 | 0.48 | 0.21 | 1.11 | 0.09 | 0.37 |
| CES-D 3 (could not get rid of mood) - More often (3-4 days) | 0.14 | 0.45 | 0.30 | 1.15 | 0.47 | 2.79 | 0.76 | 0.83 |
| CES-D 3 (could not get rid of mood) - Mostly (5-7 days) | 0.24 | 0.52 | 0.46 | 1.27 | 0.46 | 3.55 | 0.65 | 0.82 |
| Thyroid medication at baseline? Yes | -0.89 | 0.50 | -1.78 | 0.41 | 0.15 | 1.10 | 0.08 | 0.37 |
| Drink alcohol more than once a week? Yes | -0.33 | 0.36 | -0.91 | 0.72 | 0.35 | 1.46 | 0.36 | 0.67 |
| Sex - Female | 0.08 | 0.42 | 0.20 | 1.09 | 0.47 | 2.49 | 0.85 | 0.85 |
| Smoke? No, but I used to | -0.22 | 0.37 | -0.61 | 0.80 | 0.39 | 1.65 | 0.54 | 0.82 |
| Smoke? Yes | -0.19 | 0.36 | -0.52 | 0.83 | 0.41 | 1.67 | 0.60 | 0.82 |
| Number of previous hospital admissions? | 0.41 | 0.16 | 2.61 | 1.50 | 1.11 | 2.03 | 0.009* | 0.13 |
| PSQI (sleep quality index global score) | 0.28 | 0.19 | 1.48 | 1.32 | 0.91 | 1.92 | 0.14 | 0.42 |
| Mean right hippocampal volume | 0.54 | 0.21 | 2.54 | 1.72 | 1.13 | 2.62 | 0.011* | 0.13 |
| Intracranial volume | -0.18 | 0.23 | -0.79 | 0.84 | 0.54 | 1.30 | 0.43 | 0.74 |
| Total cholesterol | -0.23 | 0.15 | -1.53 | 0.80 | 0.60 | 1.07 | 0.13 | 0.42 |
| Age | 0.03 | 0.16 | 0.18 | 1.03 | 0.76 | 1.39 | 0.85 | 0.85 |
| BMI | -0.08 | 0.17 | -0.46 | 0.93 | 0.67 | 1.29 | 0.65 | 0.82 |
| Medication index | 0.15 | 0.17 | 0.93 | 1.17 | 0.84 | 1.62 | 0.35 | 0.67 |

**Supplementary Figure 1.** Mean right hippocampal volume, cholesterol, PSQI sleep quality global score, and previous number of inpatient episodes stratified by rehospitalization status. Notes: Red: Rehospitalized = True, Blue: Rehospitalized = False.

**

**

**Supplementary Figure 2.** CES-D 3: Could not get rid of my troubling mood, CES-D 5: Last week I had trouble concentrating, currently use diazepines, oxazepines, thiazepines, oxepines, and alcohol consumption stratified by rehospitalization status. Notes: Red: Rehospitalized = True, Blue: Rehospitalized = False.

**

**

**Supplementary Figure 3.** Thyroid medication and PSQI item 7 stratified by rehospitalization status. Notes: Red: Rehospitalized = True, Blue: Rehospitalized = False.**

**
